# SELF-PERCEPTIONS OF AGING MEDIATE THE ASSOCIATIONS OF CHANGE IN DEPRESSION WITH LIFE SATISFACTION AND DISABILITY

**DOI:** 10.1093/geroni/igae098.0284

**Published:** 2024-12-31

**Authors:** Serena Sabatini

**Affiliations:** University of Surrey, Guildford, England, United Kingdom

## Abstract

Self-perceptions of ageing (SPAs) significantly influence individuals’ health trajectories and satisfaction with life. This study explores (1) the associations between 14-year change in depressive symptoms as predictor of follow-up scores on SPAs, satisfaction with life, and informant-rated disability and (2) whether SPAs mediate the associations of 14-year change in depressive symptoms with follow-up scores on satisfaction with life and informant-rated disability. Participants were 174 older adults (Mean age= 87.41; SD= 3.67; 60% women) from the Sydney Memory and Ageing Study (MAS) with complete wave 7 (14-year follow-up) data. Measures used were the Geriatric Depression Scale (GDS); the informant-rated World Health Organisation Disability Assessment Schedule (WHODAS); the Diener Satisfaction with Life Sclae (SwL); and the Laidlaw’ Attitudes to Aging Questionnaire (AAQ) as indicator of SPAs. We calculated a change score for depressive symptoms. Linear regression models and structural equation models were used. After controlling for covariates of age, sex, marital status, occupation, and number of chronic health conditions, greater 14-year increase in depressive symptoms predicted more negative scores on SPAs (Standardised beta, β = 0.41; p<.001), lower SwL (β = 0.30; p<.001), and greater informant-rated disability (β = 0.26; p=.001). Participants’ scores on SPAs partially mediated the association of 14-year change in depressive symptoms as predictors of follow-up scores on satisfaction with life and informant-rated disability. Negative SPAs may be one of the reasons why increased depressive symptoms in old and very old age can be related to lower satisfaction with life and informant-rated disability.

